# Induction of Neural Plasticity Using a Low-Cost Open Source Brain-Computer Interface and a 3D-Printed Wrist Exoskeleton

**DOI:** 10.3390/s21020572

**Published:** 2021-01-15

**Authors:** Mads Jochumsen, Taha Al Muhammadee Janjua, Juan Carlos Arceo, Jimmy Lauber, Emilie Simoneau Buessinger, Rasmus Leck Kæseler

**Affiliations:** 1 Department of Health Science and Technology, Aalborg University, 9220 Aalborg, Denmark; taha@hst.aau.dk (T.A.M.J.); rlk@hst.aau.dk (R.L.K.); 2 LAMIH UMR CNRS 8201, INSA Hauts de France, Université Polytechnique Hauts de France, F-59313 Valenciennes, France; juancarlos.arceo@uphf.fr (J.C.A.); jimmy.lauber@uphf.fr (J.L.); emilie.simoneau@uphf.fr (E.S.B.)

**Keywords:** brain-computer interface, neural plasticity, neurorehabilitation, motor imagination, exoskeleton

## Abstract

Brain-computer interfaces (BCIs) have been proven to be useful for stroke rehabilitation, but there are a number of factors that impede the use of this technology in rehabilitation clinics and in home-use, the major factors including the usability and costs of the BCI system. The aims of this study were to develop a cheap 3D-printed wrist exoskeleton that can be controlled by a cheap open source BCI (OpenViBE), and to determine if training with such a setup could induce neural plasticity. Eleven healthy volunteers imagined wrist extensions, which were detected from single-trial electroencephalography (EEG), and in response to this, the wrist exoskeleton replicated the intended movement. Motor-evoked potentials (MEPs) elicited using transcranial magnetic stimulation were measured before, immediately after, and 30 min after BCI training with the exoskeleton. The BCI system had a true positive rate of 86 ± 12% with 1.20 ± 0.57 false detections per minute. Compared to the measurement before the BCI training, the MEPs increased by 35 ± 60% immediately after and 67 ± 60% 30 min after the BCI training. There was no association between the BCI performance and the induction of plasticity. In conclusion, it is possible to detect imaginary movements using an open-source BCI setup and control a cheap 3D-printed exoskeleton that when combined with the BCI can induce neural plasticity. These findings may promote the availability of BCI technology for rehabilitation clinics and home-use. However, the usability must be improved, and further tests are needed with stroke patients.

## 1. Introduction

Brain-computer interfaces (BCIs) have over the past years been proposed also as a tool for motor rehabilitation after neural injuries, such as spinal cord injury or stroke [1,2,3,4,5,6]. It is well-established that BCIs can be used for inducing neural plasticity [7,8,9,10,11], which is believed to be the underlying mechanism of motor learning/recovery [12]. These neuroplastic changes are induced in the brain by pairing the movement-related activity of the brain with the inflow of congruent somatosensory feedback from, e.g., electrical stimulation [7], rehabilitation robots, or exoskeletons [8]. Movement-related cortical potentials [13] or event-related desynchronization [14,15] have typically been extracted from single-trial electroencephalography (EEG) recordings and used as the control signals for triggering the external device that is going to elicit the somatosensory feedback. The concept of inducing plasticity using a BCI has been shown in several studies; however, this technology is rarely used in rehabilitation clinics and the patient’s home. This is due to several reasons, one of them being that it is still a fairly new technology, while some of the translational issues include the complexity of the systems in terms of setting up (e.g., mounting the EEG cap and calibrating the system), ensuring a good and stable signal quality, which may require a skilled operator, the mental fatigue of the user, user compliance, the price of the technology, and access to the detection algorithms [16,17]. In recent years, several low-cost commercial EEG systems have become available [18]. Some of these systems may not be useful for applications where neural plasticity is induced in the motor system, since they do not record electrical activity from the relevant brain areas [19]. However, it is possible to record the electrical activity of the motor cortex with some low-cost EEG systems. Moreover, several research groups have made their detection algorithms publicly available (see, e.g., the OpenViBE project [20]). The feasibility of using such low-cost systems for detecting movement-related brain activity has been outlined recently [21,22,23], thus making the BCI technology available to a wider audience than BCI researchers. To use the BCI for inducing neural plasticity, besides for neurofeedback applications [24], an external device is needed to provide congruent somatosensory feedback. This could be a stimulator that could stimulate the relevant nerves and muscles electrically, or it could be an exoskeleton. With the current advances made within the design and manufacturing of exoskeletons through 3D printing [25,26], it has become cheap to create simple exoskeletons for controlling certain joints such as the wrist or ankle. It is possible to create a simple exoskeleton that can perform wrist extensions or the dorsiflexion of the ankle joint with a single actuator [11]. Both of these movement types are important to train during stroke rehabilitation. It has been shown previously that neural plasticity, when quantified with transcranial magnetic stimulation (TMS), can be induced using BCI-triggered electrical stimulation and passive movements from rehabilitation robots/exoskeletons for the cortical projections of the lower limb muscles [7,8,9,10,11], but this has not been shown for the cortical projections of the upper limb muscles, although functional improvements in stroke patients have been reported for the upper limbs (see, e.g., Refs. [4,5,27,28]). Therefore, the aim of this study is to investigate if a BCI-triggered exoskeleton can induce neural plasticity in the cortical projections of the forearm muscles that control wrist extension. Moreover, it will be tested if this is possible using a low-cost EEG amplifier and open source BCI software. Lastly, a cheap 3D-printed exoskeleton will be developed to replicate wrist extension. The BCI-triggered exoskeleton will be evaluated in terms of BCI system performance and the ability to induce neural plasticity.

## 2. Materials and Methods

### 2.1. Subjects

Eleven healthy subjects participated (four females, age: 28 ± 3 years). Prior to participation, the subjects provided their written informed consent and filled in a questionnaire for their eligibility for TMS based on the recommendations in Ref. [29]. All procedures were approved by the local ethical committee (N-20130081), and were in accordance with the Helsinki Declaration.

### 2.2. Experimental Setup

**Figure 1:**
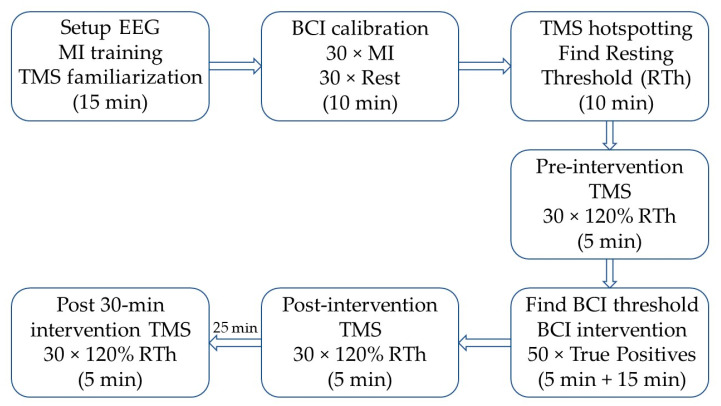
Timeline of the experiment; the approximate duration of each block is indicated in parentheses. First, the subject was familiarized with transcranial magnetic stimulation (TMS) and motor imagination (MI), and the EEG cap was mounted. Next, the brain-computer interface (BCI) was calibrated, followed by the identification of the optimal stimulation site (hotspot) and intensity (RTh). The pre-intervention TMS, post-intervention TMS, and post-30 min intervention TMS were identical. After the pre-intervention TMS, the threshold for each subject was tested with an online BCI and changed if needed. Afterwards, the intervention started, and it was stopped when the subject reached 50 correct pairings between motor imagination (MI) and movement of the exoskeleton. The post-30 min intervention TMS started 30 min after the BCI intervention ended.

Initially, the subjects were seated in a comfortable chair, where the procedures were explained, and they were familiarized with TMS. See Figure 1 for a timeline of the experiment. Afterwards, they were instructed on how to perform motor imagination, and they spent ~5 min training this. After the motor imagination training, the subjects imagined 30 wrist extensions of the right wrist while continuous EEG was recorded. A visual cue was generated by the “Motor Imagery BCI” in OpenViBE; the visual cue was modified such that 30 idle/rest trials (“REST” was displayed on the screen) and 30 motor imagination trials (a red arrow pointing to the right was displayed on the screen) were performed. The imaginary movement was maintained for four seconds. These trials were used to calibrate the asynchronous BCI for controlling the wrist exoskeleton. During the actual BCI training, the wrist exoskeleton was mounted on the subject on the right forearm and hand. The forearm and hand rested on the armrest of the chair during the training. The subjects were asked to trigger the exoskeleton by imagining an extension of the right wrist; the training was complete when 50 correct pairings of motor imagination and the movement of the exoskeleton were obtained. The subjects had to keep imagining the movement while the exoskeleton performed the movement. Before, immediately after, and 30 min after the BCI training, TMS measurements were performed, whereby 30 motor-evoked potentials (MEPs) were obtained. 

### 2.3. Recordings

#### 2.3.1. EEG

Seven channels of continuous EEG were recorded (Cyton Biosensing Board, OpenBCI, Brooklyn, NY, USA) from F1, F2, C3, Cz, C4, P1, and P2 with respect to the International 10-20 System using sintered ring electrodes placed in an EASYCAP EEG cap (EASYCAP GmbH, Herrsching, Germany). The signals were sampled at 250 Hz. The ground electrode was placed at AFz, and the reference electrode was placed on the mastoid bone behind the right ear. The subjects were asked to sit still and avoid the contraction of facial muscles and blinking. 

#### 2.3.2. EMG

MEPs were recorded using surface EMG electrodes (Neuroline 720, Ambu A/S, Ballerup, Denmark) placed on the extensor digitorum muscle in a bipolar derivation. Two electrodes were placed on the belly of the muscle, which was identified through palpation, and a ground electrode was placed on the distal head of the Humerus bone. The signals were amplified with a gain of 5000 using a customized amplifier (Jan Stavnshøj, Aalborg University), and the signals were sampled at 4000 Hz using the Mr. Kick software (Knud Larsen, Aalborg University). 

### 2.4. Transcranial Magnetic Stimulation

**Figure 2:**
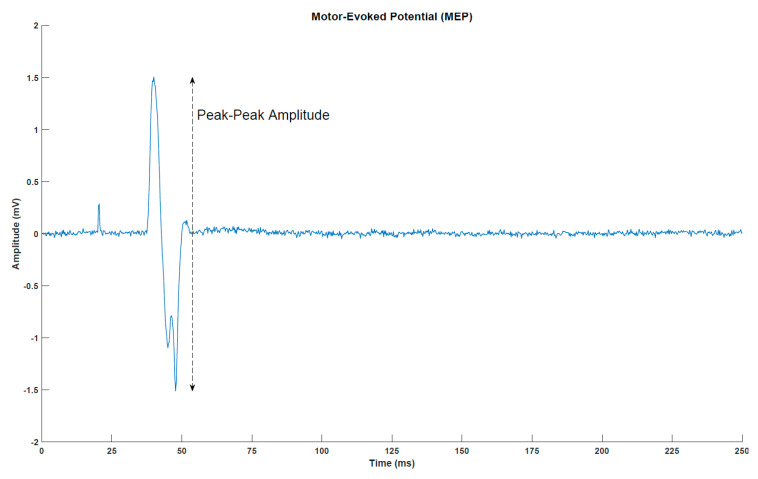
Motor-evoked potential (MEP) from a representative subject (post-intervention transcranial magnetic stimulation measurement for subject 1). The peak around 25 milliseconds is the stimulation artefact from the transcranial magnetic stimulation.

MEPs (Figure 2) were elicited with a single-pulse TMS (Magstim 200, Magstim Company, Dyfed, UK) using a figure-of-eight coil with a posterior–anterior current direction. First, the optimal stimulation site was determined. This was defined as the location where the largest MEP peak-peak amplitudes were obtained. Next, the resting threshold was determined. It was defined as the lowest stimulation intensity that would elicit an MEP of at least 50 µV peak-peak amplitude in five out of ten simulations. In the measurements before, immediately after and 30 min after the intervention, 30 stimuli were given at 120% of the resting threshold. A random break of 5–7 s separated two consecutive stimuli.

### 2.5. Brain-Computer Interface

**Figure 3:**
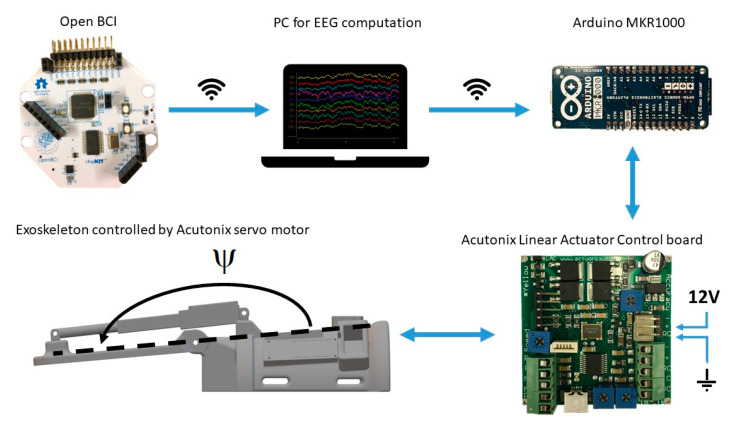
Overview of the hardware setup. The Arduino and Linear Actuator Control board were mounted on the exoskeleton. The EEG electrodes were connected through wires to the Open BCI board from which the signals were transmitted through wireless communication to the PC running OpenViBE. Once an imagined wrist extension was detected a trigger was sent through wireless communication to the Arduino on the exoskeleton. The Arduino was connected to the Linear Actuator Control board with a wire. The Linear Actuator Control board was powered with a 12 V power supply. The motor was connected to the Linear Actuator Control board with a wire.

The “Motor Imagery BCI” from OpenViBE was used for the detection of the imaginary wrist extensions. The data were bandpass filtered from 8 to 30 Hz using a 5th order Butterworth filter, and a common spatial pattern spatial filter was calculated from the calibration data, which maximized the differences in spectral power between the idle and motor imagery classes. The trials were divided into 1 s windows with a shift of 1/16 s, and the logarithmic band powers were calculated from each window and used as features. Based on the features extracted from the training data, a linear discriminant analysis classifier was trained using 5-fold cross-validation. In the online test, the classification of the imagined movement was altered compared to the original OpenViBE scenario. An imagined movement was detected when eight consecutive windows (8/16 s = 0.5 s) exceeded a threshold that was determined for each subject individually. The determination of the threshold took less than five minutes (see Figure 1), and it was done to obtain a trade-off between true positive and false-positive detections. When the BCI detected an imagined movement, a trigger was sent through a transmission control protocol to an Arduino MKR1000 that activated the wrist exoskeleton (see Figure 3). The performance metrics of the BCI were the true positive rate, the number of false negatives per minute, and the number of false positive detections per minute. The subjects indicated verbally if the trial was correct (true positive) or if it was incorrect (false positive or false negative). 

### 2.6. Exoskeleton

**Figure 4:**
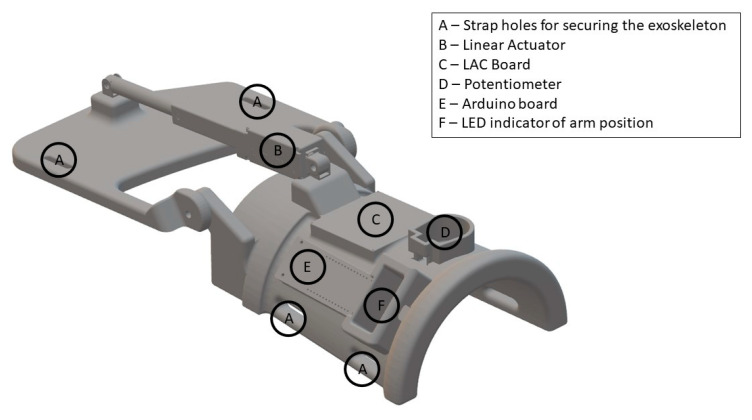
View of the 3D-printed exoskeleton. The illustration is not drawn to scale. The surfaces that were in contact with the forearm and hand were padded with foam to improve the comfort. The exoskeleton was fixated to the subject’s hand and forearm with Velcro straps (A). ‘LAC’: Linear Actuator Control.

The exoskeleton was 3D-printed and developed specifically for this study (see Figure 4). The purpose of the exoskeleton was to control the wrist angle position (denoted by Ψ) to replicate an extension of the wrist (see Figure 3). The exoskeleton was 3D-printed using the material PLAMAX, and it was actuated by a model L16-P linear piston (Actuonix, Motion Devices Inc^®^, Victoria, BC, Canada) which was connected to the X2 input of a linear actuator control (LAC) board (Actuonix, Motion Devices Inc^®^, Victoria, BC, Canada) with default settings. A 12 V power supply was connected to the piston and LAC board in the (±) X6 inputs. The analog output (A0) and ground reference of the Arduino MKR1000 were connected to the (VC) and (−) X6 inputs of the LAC board, respectively; the Arduino board was programmed and powered via a USB connection (5 V power supply). Finally, the design includes an LED bar that indicated the wrist angle position, which was connected to the (±) X3 inputs and (P) X4 input of the LAC board. The exoskeleton received an activation signal from the BCI through serial communication to the Arduino board with a baud rate of 9600. The Arduino sends a reference position signal for the linear piston and the LAC board for compensating for the position error signal of the position in order to follow the desired trajectory. The predefined trajectory Ψ_d_(t) begins at the initial position Ψ_d_(0) = 180°, then the wrist is extended in 1.8 s to Ψ_d_(1.8) = 112.36° and stays in this position for 0.5 s before returning to the initial position, which also takes 1.8 s. The average movement speed while moving is 37.58°/s. 

### 2.7. Statistics

A one-way repeated measure analysis of variance (ANOVA) with time as a factor (3 levels: pre-, post-, and post-30 intervention) was performed on the median MEP peak-peak amplitudes to investigate if there was a difference between the MEP amplitudes at the three different time points. A significant test statistic was followed up with a posthoc analysis using Bonferroni correction to avoid multiple comparisons. Moreover, Spearman correlation was calculated between the BCI performance metrics (true positive rate, the number of false negatives per minute, and the number of false positive detections per minute) including the duration of the training and the changes in MEP amplitude. Moreover, the correlation between the MEP changes and BCI performance metrics was calculated with respect to age (Spearman correlation) and gender (Point Biserial correlation). A significant test was assumed when *p* < 0.05. 

## 3. Results

**Figure 5:**
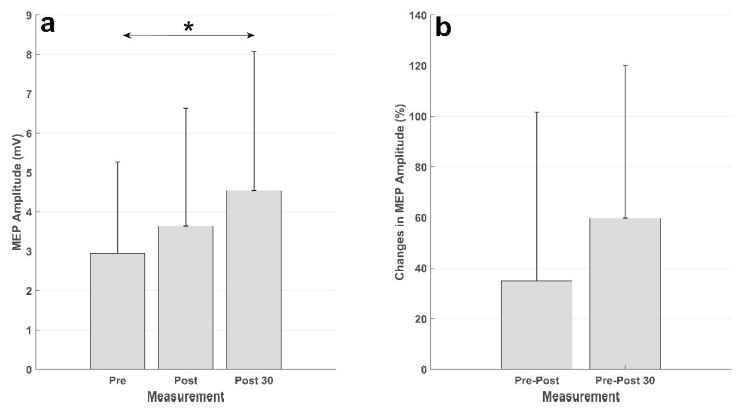
Summary of the MEP results. (**a**) Averaged MEP amplitudes (in mV) across the subjects, the vertical black line represents the standard deviation across subjects. The MEPs from the measurement 30 min after the intervention (Post 30) were significantly higher (denoted by *) than those from the measurement before the intervention (Pre). (**b**) MEP changes (in percent) from the measurement before the intervention to the measurement immediately after the intervention (Pre-Post) and 30 min after the intervention (Pre-Post 30).

The results are summarized in Table 1 and Figure 5. The presented *p*-values for the posthoc test have been Bonferroni corrected. On average, 86 ± 12% of the imaginary wrist extensions were correctly detected by the asynchronous BCI, while there were 1.20 ± 0.57 false positive detections per minute and 0.63±0.58 false negatives per minute (see Table 1). It should be noted that there is a large standard deviation, especially for the true positive rate, and especially subject 2 had difficulties in activating the exoskeleton through the BCI. The true positive rate was higher for subjects 5 and 6, but they had a large number of false positive detections per minute. 

The results of the intervention are presented in Figure 5. There was an increase in the MEP from before intervention to immediately after, and 30 min after, the intervention in both absolute units (mV) and relative units (percent). The statistical analysis showed a significant effect of time (F _(2,20)_ = 4.63; *p* = 0.022). The posthoc analysis revealed a significant increase in the MEP amplitude from the measurement before the intervention to the measurement 30 min after the intervention (*p* = 0.028). There was no difference between the MEP from the measurement before the intervention and that immediately after (*p* = 0.73), or the MEPs in the measurements after the intervention (*p* = 0.34). 

There was no correlation between the true positive rate (correlation coefficient: 0.36; *p* = 0.28), the number of false positive detections per minute (correlation coefficient: −0.35; *p* = 0.30), the number of false negatives per minute (correlation coefficient: −0.39; *p* = 0.24), or duration (correlation coefficient: 0.31; *p* = 0.36), and the changes in MEP amplitude from before to 30 min after the intervention. Age and gender did not correlate with any of the other measures.

## 4. Discussion

It was possible to detect imaginary wrist movements with a low-cost BCI with a true positive rate of 86 ± 12%, with 1.20 ± 0.57 false detections and 0.63 ± 0.58 false negatives per minute. The BCI training with the exoskeleton led to increased MEPs after the training with respect to the pre-intervention measurement. There was a non-significant increase from pre- to post-intervention measurements of 35 ± 60%, and a significant increase from pre- to post-30 min intervention measurements of 67 ± 60%.

### 4.1. Induction of Plasticity 

The BCI-triggered exoskeleton movements increased the excitability of the cortical projections to the forearm extensor muscles. The increase in MEP size was similar to what has been reported previously for the electrical stimulation of the radial nerve based on an associative BCI, which was approximately 50% compared to baseline MEPs [30]. In addition, the changes in excitability are in a similar range of what has been reported previously for BCI-triggered electrical stimulation of the common peroneal nerve and exoskeleton movement of the ankle joint. The BCI intervention in these studies has consistently reported increases in corticospinal excitability in the range of 40–100% [7,8,9,10,11,31]. In this study, the increase from pre- to post-intervention measurement was not significant, however, it could be attributed to the large standard deviation of approximately 60%, or to the fact that the effect of the intervention takes some time to consolidate. Large variability has commonly been reported in neuromodulation studies where the effect of the intervention was quantified using MEPs elicited through TMS [7,8,9,10,11,32]. The variability in the MEP size is affected by multiple factors, such as attention and time of day (reviewed in Refs. [33,34]), but it may also be due to the variable response of neuromodulation interventions [35,36,37,38]. It should also be noted that there exist other types of techniques that have been used for the induction of neural plasticity and proposed for stroke rehabilitation, and that activate the cortical brain areas [39]. One of these techniques is repetitive TMS, whereby the cortical excitability of specific brain areas can be upregulated, which has led to increased amplitudes of motor-evoked potentials [40]. TMS has also been paired with afferent inflow from the electrical stimulation of a peripheral nerve (paired associative stimulation). This protocol has been used to consistently induce neural plasticity when the correct interstimulus interval between the magnetic and electrical stimulation has been selected [33]. However, the use of TMS may not be tolerated well by some stroke patients [41], and there would be safety precautions to consider [29]. Another way to activate the motor cortex is by the use of transcranial direct current stimulation, which has been used to increase the excitability in the motor cortex [42,43,44]. Moreover, this technique has been used for priming before BCI training, but there is no clear indication of an additive effect [45,46]. However, it has been shown that transcranial direct current stimulation can improve the BCI performance [45], possible through the modulation of the mu event-related desynchronization [47]. 

The induction of plasticity in this study is expected to be due to the combination of motor imagery and afferent feedback that was temporally correlated. Two control experiments could have been performed to investigate the effect of motor imagery alone on the MEP and the effect of passive movement alone on the MEP amplitude. However, the control experiment for motor imagery has been conducted three times, wherein 50 imaginary movements have been performed, and no change in MEP amplitudes has been reported [7,8,48]. For the afferent feedback alone (i.e., passive movement in this study), it has been reported that 50 passive movements do not change the MEP amplitudes [8], and when delivering afferent feedback through electrical stimulation alone (50 stimuli), no change in MEP amplitudes has been reported [7,49]. The changes in plasticity in this study are expected to be mediated through long-term potentiation (LTP)-like changes, as has been suggested in several previous studies using a similar methodology [8,9,48]. The criteria of LTP-like plasticity include associativity (pairing between motor imagery and afferent feedback from the passive movement), rapid onset (indicated by the post-intervention measurement), and lasting effects (at least 30 min, as indicated by the post-30 min intervention measurement) [50]. There was only a measurement 30 min after the intervention, but the changes associated with this intervention have been reported to last at least 60 min [51]. It is possible that the effects last longer, but probably not longer than 24 h. It has been reported that there was no difference in MEP amplitude between two pre-intervention measurements before two similar plasticity-inducing protocols, when separated by 24 h [49]. Another criterion for LTP-like plasticity is specificity, which was not tested in this study, but it has been reported that this type of intervention is specific [48]. The changes in the neural plasticity that were observed could happen throughout the nervous system, but it has been suggested in several studies using the stretch reflex that the changes are supraspinal [7,8,32,48]. 

### 4.2. Brain-Computer Interface System Performance 

The BCI system that was used in this study performed well in terms of the true positive rate and number of false positive detections per minute. The performance is comparable with other asynchronous BCI studies that have been used for inducing plasticity, which have reported true positive rates in the range of 67–85% and a number of false positive detections per minute in the range of 0.5–2.8 [5,7,8,9,10,11,31]. The approaches to movement intention detection in those studies have primarily relied on movement-related cortical potentials, but the results of the current study show that a BCI based on sensorimotor rhythms is just as effective in terms of movement intention detection, and it has also been used successfully for BCI training in stroke patients [4,5,52,53]. The BCI performance of the participants in this study was variable, but there was only a single participant that experienced low control (true positive rate of 56%). It would be possible to reduce the detection threshold to allow a higher true positive rate, but that also increases the number of false-positive detections per minute. However, the number of false-positive detections could potentially be controlled using a paradigm whereby the BCI only accepts inputs in predefined periods, instead of always being active, as in the asynchronous paradigm, or if the number of windows is increased when the detection is exceeded; the latter approach would increase the detection latency. It has been shown previously that the afferent feedback should coincide with the movement intention (i.e., short detection latency) [48], but recent findings have suggested that plasticity can be induced with less strict detection latencies [9]. This may allow the use of residual EMG, from which it is possible to decode multiple movement types [54], which could introduce some task variability in the training [55], and it may be easier for the stroke patients to control the exoskeleton. The correlation analysis showed that there was no correlation between the induction of plasticity in terms of peak-peak amplitudes in the MEP and the performance metrics of the BCI system. This may suggest that the current level of BCI system performance is sufficient for inducing plasticity, and that it may not be needed to optimize the system further from the movement detection point of view, although it should be pointed out that correlation analyses were performed on a limited sample wherein all subjects (except one) had good performance. It has been reported previously that the true positive rate, number of false positive detections and total time of the intervention explain little of the variance in the peak-peak amplitude of the MEP, with the duration explaining more than the other two measures [9,11]. However, in a similar BCI study, Niazi et al. reported a statistically significant correlation of 0.8 between the BCI system’s performance and changes in MEP amplitude in eight healthy subjects [7]. The BCI system performance in that study was calculated as the ratio between the true positive rate and false positive detections; when performing the same calculation in the current study, a similar significant correlation is observed (correlation coefficient: 0.64; *p*=0.034). This indicates that there is an incentive to improve the BCI performance. 

### 4.3. Limitations and Future Perspectives 

In this study, it was shown that young healthy participants could control the BCI, and it could be used for inducing neural plasticity. These findings should be validated in future studies with the intended end-users, which are stroke survivors with motor impairment who often are more than 65 years old. The motor cortex excitability decreases with age [34], but it has been shown that the MEP amplitudes can increase 100% in stroke patients using an associative BCI protocol [56]. It is likely that the reported BCI system’s performance will be slightly lower for stroke patients [13,57,58], and if the same experimental protocol is used, it must be considered that some of the stroke patients may not be able to communicate due to, e.g., aphasia, and hence will not be able to indicate what they intended to do (i.e., true positive, false positive or false negative). An alternative approach to verbal feedback to the experimenter could be to use error-related potentials as a check to indicate if the trial was a true positive or false positive; however, this approach will also be prone to the uncertainty related to the decoding algorithm used for these potentials [59]. The lower BCI system performance for stroke patients may cause frustration, and the performance can be affected by fatigue and inattention. To avoid this, it could be a possibility to implement the BCI training in a game to make the training more engaging. Using game mechanics, it would be possible to bias the classifier to improve the performance and conceal it for the user. The proposed system may be used for motor training in this patient group, but it is important to note that an increase in MEP size does not equal improvement in motor function, although increases in MEP size have been reported alongside skill acquisition in healthy participants [12] and motor recovery in stroke patients [56,60]. The BCI training may be used as a training intervention in itself, but it could also be possible to utilize the lasting increase in the MEP size (more than 30 min) in a rehabilitation scenario where the BCI training is used to prime the nervous system before other types of training, such as physiotherapy or occupational therapy. For the BCI to be used in rehabilitation clinics or the home of the patient, the usability should be improved in terms of various aspects, such as the hardware setup, which should be simplified, and the safety of BCI use in acute patients should be assessed [61]. The BCI system should be coded on the Arduino to reduce the amount of hardware, eliminate potential communication problems and delays, which could allow a faster response of the exoskeleton, and the calibration time should be reduced or removed using, e.g., a subject-independent movement intention detector [62,63]. As regards the communication problems, the robustness of the EEG recordings and usability testing is where the expensive systems potentially differ most from the current cheaper alternatives (this is just a speculation); these are important factors to consider for the technology to be adopted in a clinical setting. It should be investigated if the cost of the BCI (i.e., the EEG amplifier) and exoskeleton can be reduced further. In total, the price for the BCI system (including EEG amplifier, cap, electrodes and cables) and the exoskeleton (all parts including motor, control board and Arduino) was less than USD 1000. Additionally, the design of the exoskeleton should be improved so it will be easier to put on and take off by the users, and the comfort should be increased as well. This could be done by adapting the exoskeleton to the individual, which would be possible when the components are 3D-printed. Another option could be to use soft exoskeletons, such as a glove or sleeve, that can perform the intended movements [64]. 

In this study, the exoskeleton was used to execute the intended movement and provide afferent feedback, but it would be possible to use electrical stimulation as well to provide the afferent feedback. In two recent studies, it has been shown that there is no difference between the afferent feedback from electrical stimulation and passive movements from an exoskeleton/robot in terms of the induction of plasticity [11,32]. This gives the patient and therapist freedom to choose the modality that works best for the patient. Some patients may not be able to tolerate electrical stimulation well, or may have problems in placing the stimulation electrodes correctly, so the usability of the exoskeleton may be better compared to electrical stimulation; however, this has not been tested, and it should be validated in future studies with the end-users.

To summarize, for this technology to be used in a home setting the BCI and exoskeleton have to fulfill some requirements, as follows: (1) it must be easy to take on and off the exoskeleton; (2) the software must be easy to use, since the user may not be used to working with technology and may suffer from cognitive impairments to some degree; (3) the system calibration should be done automatically; (4) the hardware setup of the BCI must be simple, and (5) the patient should be able to place EEG electrodes over the motor cortex. The latter may be difficult for patients with severe motor impairments; in a recent study it was shown that half of the stroke participants were able to mount EEG headsets that covered the motor cortex while the other half was unable to mount the EEG headsets [17]. Those that could mount the headset spent roughly 10 min. On the contrary, relatives to the patients and therapists could quickly mount the EEG headset with little prior instruction (5 min). The setup times for them were between 3 and 5 min. Thus, it would be important to have a headset that is easy to mount with one hand for the most affected patients, unless they have someone to help them with the setup. 

## 5. Conclusions

In this study, it was shown that the movement intentions associated with imagined wrist extensions could be detected with good performance (true positive rate: 86 ± 12%; number of false positive detections per minute: 1.20 ± 0.57) using a cheap amplifier and open source BCI system. A cheap 3D-printed wrist exoskeleton was developed, wrist extensions were successfully replicated, and afferent feedback was provided. The BCI-triggered exoskeleton movements increased the excitability of the cortical projections to the extensor muscles in the forearm. These findings may have implications for the transfer of BCI technology to rehabilitation clinics and home training, by making the technology affordable to more rehabilitation clinics and patients. 

## Figures and Tables

**Table 1:** Brain-computer interface performance.

Subject	True Positive Rate (%)	False Negatives per Minute	False Positive Detections per Minute	Duration of the Training (Minutes)
1	93	0.36	0.55	11
2	56	2.11	0.78	18
3	98	0.10	1.00	11
4	79	1.08	0.50	12
5	81	1.09	2.10	11
6	83	0.67	1.93	15
7	100	0	1.81	16
8	94	0.23	1.77	13
9	86	0.53	1.10	15
10	89	0.43	0.57	14
11	94	0.33	1.11	9
Mean ± std	86 ± 12	0.63 ± 0.58	1.20 ± 0.57	13 ± 3

## Data Availability

The data presented in this study are available on request from the corresponding author.
